# Contagion of Cooperation in Static and Fluid Social Networks

**DOI:** 10.1371/journal.pone.0066199

**Published:** 2013-06-19

**Authors:** Jillian J. Jordan, David G. Rand, Samuel Arbesman, James H. Fowler, Nicholas A. Christakis

**Affiliations:** 1 Department of Psychology, Harvard University, Cambridge, Massachusetts, United States of America; 2 Institute for Quantitative Social Science, Harvard University, Cambridge, Massachusetts, United States of America; 3 Ewing Marion Kauffman Foundation, Kansas City, Missouri, United States of America; 4 Department of Psychology, Yale University, New Haven, Connecticut, United States of America; 5 Department of Sociology, Yale University, New Haven, Connecticut, United States of America; 6 Political Science Department, University of California San Diego, La Jolla, California, United States of America; 7 Medical Genetics Department, University of California San Diego, La Jolla, California, United States of America; University of Zaragoza, Spain

## Abstract

Cooperation is essential for successful human societies. Thus, understanding how cooperative and selfish behaviors spread from person to person is a topic of theoretical and practical importance. Previous laboratory experiments provide clear evidence of social contagion in the domain of cooperation, both in fixed networks and in randomly shuffled networks, but leave open the possibility of asymmetries in the spread of cooperative and selfish behaviors. Additionally, many real human interaction structures are dynamic: we often have control over whom we interact with. Dynamic networks may differ importantly in the goals and strategic considerations they promote, and thus the question of how cooperative and selfish behaviors spread in dynamic networks remains open. Here, we address these questions with data from a social dilemma laboratory experiment. We measure the contagion of both cooperative and selfish behavior over time across three different network structures that vary in the extent to which they afford individuals control over their network ties. We find that in relatively fixed networks, both cooperative and selfish behaviors are contagious. In contrast, in more dynamic networks, selfish behavior is contagious, but cooperative behavior is not: subjects are fairly likely to switch to cooperation regardless of the behavior of their neighbors. We hypothesize that this insensitivity to the behavior of neighbors in dynamic networks is the result of subjects’ desire to attract new cooperative partners: even if many of one’s current neighbors are defectors, it may still make sense to switch to cooperation. We further hypothesize that selfishness remains contagious in dynamic networks because of the well-documented willingness of cooperators to retaliate against selfishness, even when doing so is costly. These results shed light on the contagion of cooperative behavior in fixed and fluid networks, and have implications for influence-based interventions aiming at increasing cooperative behavior.

## Introduction

Cooperation occurs when individuals pay costs to benefit others, and this behavior is a central component of human societies [1]–[14]. Evidence suggests that people are influenced by their social contacts, and that, as a result, emotions, ideas, and behaviors can spread across social network ties [15]–[28]. The question arises, therefore, of whether cooperation also spreads via social contagion. This question is theoretically interesting, and also has practical applications for interventions designed to promote cooperative behaviors.

Answering this question can be difficult, however, as contagion is often hard to disentangle from homophily (the tendency for people to form and maintain connections with similar others) [23], [29], [30]. It is particularly hard to differentiate between contagion and homophily using observational data, as homophily may occur on unobserved traits for which conventional statistical control is not possible in network analysis, as in all other observational settings [30], [31]. Controlled laboratory experiments, however, address these concerns: in the lab, experimenters have complete information about (and control over) the interaction structure of their subjects.

Leveraging the power of laboratory experiments, a recent study [32] demonstrated that social contagion does indeed occur in the context of cooperation. We used data from a classic laboratory experiment involving a public goods game (a game theoretic formalization of group social dilemmas) to examine social contagion of cooperation. In the experiment, subjects were randomly assigned to interact with new groups of strangers in each round, and chose how much money to contribute to a common project that benefited all group members. By giving subjects no control over their interaction partners, the possibility of homophily was excluded. Nonetheless, subjects who were assigned to groups of relatively high contributors made higher contributions in subsequent rounds. This result provides clear evidence for the contagion of contribution behavior in random networks with anonymous strangers in a laboratory setting with no homophily.

Evidence from other controlled laboratory experiments suggests that behavior in cooperation games is also contagious in fixed social networks, in which homophily is likewise excluded because subjects are assigned to play with the same partners each round. Across three similar experiments [33]–[35], subjects played multi-player, repeated prisoner’s dilemma games in fixed networks of various structures. In the prisoner’s dilemma, cooperation is measured through a binary choice to cooperate or defect. Thus, unlike in the public goods game, where cooperation is measured as a continuous variable, these games allow one to separately evaluate if cooperative and selfish behaviors are contagious. Across all three experiments, cooperators who were paired with relatively more defecting neighbors were more likely to switch to defection in subsequent rounds; that is, selfish behavior was contagious. However, cooperative behavior was not: defectors who were paired with relatively more cooperative neighbors were not more likely to switch to cooperation. These experiments provide further evidence that social behavior in the context of cooperation can spread from person to person, and extend this result to fixed networks. They also suggest that there may be asymmetries in the extent to which cooperative and selfish behaviors are contagious.

In these fixed network experiments, subjects were informed not only of each of their neighbors’ decisions, but also of each neighbor’s resulting total payoff. Given that defectors tended to outperform cooperators, this payoff information may have undermined the spread of cooperation: defectors with many cooperative neighbors may have felt motivated to switch to cooperation, but then have overridden this desire in the face of the knowledge that switching would reduce their payoff. Thus, more research is needed to determine if cooperative behavior may be contagious in the absence of payoff information.

Furthermore, there is a need to explore the spread of cooperation in *dynamic* rather than fixed networks. In many social environments, humans have control over their interaction partners, and are free to cut old ties and form new ones. A wider range of strategies are available in such dynamic networks compared to static networks. Thus, the strategic settings of fixed and fluid networks may promote different approaches to behavioral updating; that is, the contagion of cooperative and selfish behaviors may operate very differently in fixed and fluid networks. When humans interact repeatedly in relatively fixed social networks, a primarily goal may be to balance the competing desires of (i) successfully cooperating with others (as mutual cooperation is preferable to mutual defection) and (ii) avoiding exploitation by free-riders (as defecting with a defector is preferable to cooperating with a defector). A common solution to this dilemma is to use reactive strategies; that is, to respond to the behavior of your interaction partners by cooperating when they are cooperative and defecting when they are not. Thus, in repeated cooperation games, people tend to either defect unconditionally or play conditional strategies (such as Tit-for-tat or Grim) [36]–[41]. For example, consider Tit-for-tat, a strategy for repeated games in which a player chooses the option her opponent chose in the previous round [2]. Humans may implement these conditional strategies by mimicking their neighbors’ behavior over time. On this basis, we predict that cooperative and selfish behaviors should be contagious in relatively fixed social networks.

In contrast, in fluid social networks, another goal exists: attracting new cooperative interaction partners. If individuals (correctly) believe that they are more likely to form connections with cooperators when they themselves cooperate, they may be motivated to try cooperation even when their current interaction partners are relatively uncooperative. Thus, we might predict less of a relationship between the behavior of one’s current neighbors and one’s own future behavior in rapidly updating social networks where there is a sizable opportunity to attract new cooperative partners.

Here, we test these predictions empirically by asking how the spread of cooperative and selfish behaviors in social networks depends on the extent to which individuals have control over their network connections. We explore this issue using data from a controlled laboratory experiment examining binary group cooperation decisions in repeated games. In this experiment, networks varied in the extent to which individuals had control over their network connections. This dataset provides complete information about the history of network connections, allowing us to take a longitudinal approach to isolate social contagion across time even when homophily (based on network updating) is actually possible. Additionally, in this experiment, participants did not receive information about their neighbors’ payoffs each round, removing this potential confound for studying contagion. We use this dataset to ask how cooperative and selfish behaviors spread over time in social networks with different rules guiding their structural evolution.

## Materials and Methods

### Ethics Statement

This research was approved by the committee on the use of human subjects in research of Harvard University. Written informed consent was obtained from all subjects.

### Procedure

To examine contagion of cooperation in networks in which individuals have more or less control over their network connections, we take advantage of a dataset from a previously published experiment on cooperation in dynamic social networks [42]. In this study, subjects recruited from Amazon Mechanical Turk [43]–[48] engaged in a stochastically repeated social dilemma on a social network in groups averaging 20 people in size. At the beginning of each session, subjects were assigned to positions on social networks, with 20% of possible links between players formed at random. Then, in each round, subjects chose between cooperating, which entailed paying 50 points per neighbor for each neighbor to receive 100 points, or defecting, which entailed paying no costs and generating no benefits for any neighbors. Before making their decisions, subjects were reminded of their total number of neighbors, and (in all rounds other than the first) they were also reminded of the decisions of each neighbor in the previous round. After making their decisions, subjects were informed of their resulting payoff and the decisions of each of their neighbors. At the end of every round, there was a 0.8 probability of playing another round, and a 0.2 probability of the experiment ending. An important feature of this design is that subjects made only a single binary choice of cooperation or defection each round; subjects could not choose to selectively cooperate with some neighbors but not others. Thus, subjects were faced with a group cooperative dilemma similar to a public goods game [1].

The data from this study are of use to us because they allow us to contrast behavior in networks in which individuals do and do not have control over their network ties. In the *fixed* condition, links were not updated; in all rounds, subjects played with the same neighbors. Thus, in this condition, subjects had no control over their neighbors.

In two other conditions, however, subjects had the opportunity to alter their network connections. In the *viscous* condition, subjects had a relatively limited ability to do so, and in the *fluid* condition, subjects had relatively more control. After each round where subjects chose to cooperate or defect, 10% of subject pairs in the viscous condition and 30% of pairs in the fluid condition were randomly selected. Then, one randomly selected member of each pair was informed of the other member’s behavior in the preceding cooperation round and was offered the opportunity to alter their connection with that individual. If a link did not already exist between the pair, the subject could form a new connection; if a link already existed between the pair, the subject could break the existing connection. After each network update round, subjects were informed of the total number of individuals that formed new links with them and that broke existing links with them. Thus, this updating process gave players in the viscous and fluid conditions some level of control over their network connections and some feedback about the effect of their behavior. The experiment also featured a fourth random network control condition, but for brevity we do not discuss it in this paper, as it does not add external validity over previous work.

Previous analyses of our dataset revealed that dynamic networks promote cooperation [42]. In the fixed and viscous network conditions, cooperation significantly decreased over time, but in the fluid network condition, cooperation remained robust and stable over time (as quantified statistically by a significant interaction between round number and a fluid condition indicator, in a logistic regression predicting cooperation). As a result, in later rounds, there was significantly more cooperation in the fluid than the fixed and viscous conditions. These analyses reveal that giving individuals control over their network connections promotes cooperative behavior. Mechanistically, it was shown that, in the fluid condition, cooperation was incentivized because cooperators were more likely to form and maintain beneficial connections with other cooperators. However, this incentive was insufficient to promote cooperation in the viscous connection, where only 10% of pairs per round could update their connections.

**Figure 1:**
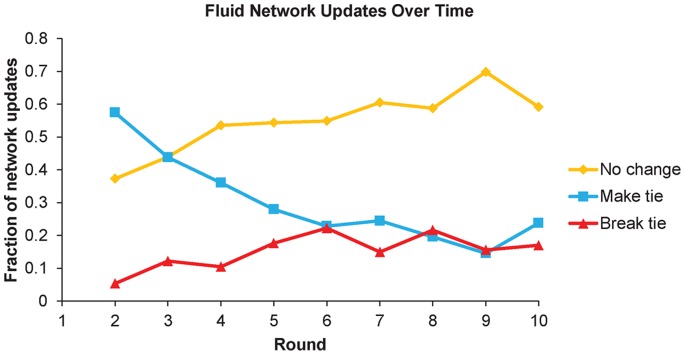
Fluid network updates over time. This figure shows the fraction of network update events in the fluid condition resulting in the formation of a new tie, the breaking of an existing tie, or no change to the network.

Additionally, previous analyses of our data set revealed that tie structure in the fluid network equilibrated over time, as shown by Figure 1 (reproduced from the Supplementary Information of ref [42]). In particular, the probability of a network update opportunity resulting in the formation of a new link declined over time. Initially, updates were much more likely to result in the formation of new links than the breaking of existing links. Eventually, however, the network stabilized into a dynamic equilibrium in which the most likely outcome was no update, and new links were formed approximately as frequently as existing links were broken (this equilibration occurred by round 5). Thus, networks in the fluid condition started out dynamic, with players frequently forming new connections, but grew less fluid over time, in actuality, as the network equilibrated.

### Analysis

In the current analyses, we examine social contagion by measuring changes in behavior across time. We examine the probability that an individual changed behavior between the previous round and the current round, as a function of her neighbors’ behavior in the previous round. We examine transitions both from cooperation to defection, and from defection to cooperation, and relate these transition probabilities to the fraction of cooperating neighbors in the previous round. This approach to measuring social contagion focuses on changes in behavior rather than frequencies of the behaviors themselves, and was adapted from previous work employing an infectious disease framework to study social contagion [27], [28]. Additionally, in light of the observation that the fluid network becomes less dynamic over time, we also look for changes in the contagion of cooperation and defection as rounds progress.

To operationalize neighbors’ behavior, we consider neighbors from the previous round, as well as, in the viscous and fluid conditions, neighbors who were added after the previous round. After subjects formed new links, they were informed of their new neighbors’ behavior in the previous round. Thus, we should expect subjects to respond to the previous behavior of both their previous neighbors and their new neighbors.

Our analysis focuses on the percentage of a subject’s neighbors that cooperated, rather than the absolute number of cooperating and/or defecting neighbors (which are distinct measures due to degree heterogeneity). We take this approach because of previous evidence suggesting that percentage of cooperating neighbors is the salient factor to subjects in cooperation experiments. Specifically, in a repeated public goods game experiment, reducing the accuracy of information subjects had about the individual decisions of other players, while continuing to accurately report the average cooperation level of the group, had no significant effect on cooperation behavior [49]; thus it seems that subjects attend to the average level of contribution (i.e. frequency of cooperation), rather than the number of cooperating neighbors.

### Statistical Methodology

Our statistical analyses use logistic regression to predict cooperation (0 = D, 1 = C) as the dependent variable, with the percentage of cooperative neighbors in the previous round as the independent variable. To examine the contagion of cooperation, we restrict to subjects who defected in the previous round (thus their cooperation decision in the current round indicates whether they stayed with D or switched to C). To examine the contagion of defection, we restrict to subjects who cooperated in the previous round (thus their cooperation decision in the current round indicates whether they stayed with C or switched to D).

We also include controls for round number, as cooperation may tend to decrease over time, and the number of neighbors an individual had in the previous round and in the current round, as the cost of cooperation increases with the number of neighbors. We use robust standard errors clustered on subject and experimental session to account for the non-independence of observations from the same subject or from subjects within the same session. Our regressions that consider changes in contagion over time also include interactions between percentage of cooperative neighbors and round number. Additionally, our regressions combining data from all three conditions include a binary ‘dummy’ variable indicating membership in the fluid condition (0 = not fluid condition, 1 = fluid condition). Because the experiment was conducted online, some subjects dropped out before their session ended; for these subjects, we analyzed all decisions they made before dropping out.

### Predictions

We predict that in the fixed and viscous conditions, subjects will mimic their neighbors’ behavior in order enact conditional cooperation strategies. Thus, cooperative and selfish behaviors should be contagious in these conditions. In contrast, we predict that when subjects in the fluid condition perceive the opportunity to attract new cooperative neighbors, they will be motivated to cooperate regardless of their neighbors’ behavior, resulting in a deflated contagion of cooperative and selfish behavior. However, this should only be true when the network is in its fluid phase (i.e. when new links are formed frequently). Thus, we predict that subjects in the fluid condition will be relatively insensitive to the behavior of their neighbors in early rounds, but that over time (as the network equilibrates) they will become more likely to imitate the defection and cooperation of their neighbors.

## Results

### Contagion of Cooperation

**Figure 2:**
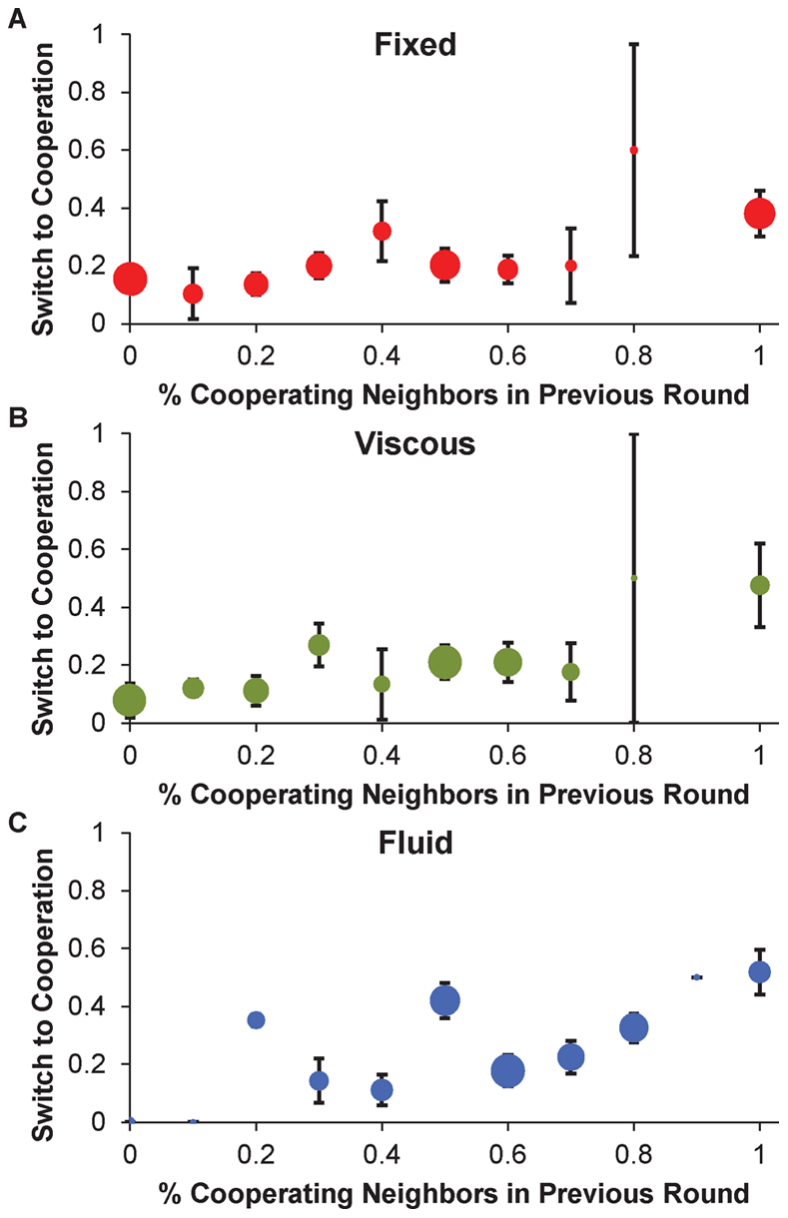
The contagion of cooperation. This figure shows the probability of switching from defection to cooperation as a function of the percentage of neighbors that cooperated in the previous round, in the (a) fixed (b) viscous and (c) fluid conditions. Dots depict the percentage of individuals with the specified range of cooperating neighbors that switched to cooperation**,** and dot size is proportional to the number of observations. Error bars indicate standard error of the mean.

We first consider the spread of prosocial behavior. Figure 2 shows, for each condition, the probability of switching from defection to cooperation as a function of the percentage of cooperating neighbors in the previous round, and Table 1 shows the accompanying regressions. We find a significant positive relationship between the probability of switching to cooperation and the percentage of cooperating neighbors in each condition individually, as well as collapsed across conditions (fixed coeff = 0.81, *p* = .015; viscous coeff = 1.50, *p* = .024; fluid coeff = 1.48, *p* = .003; all conditions coeff = 1.25, *p*<.0001; Table 1 Columns 1 through 4). Thus, across all rounds of the game, cooperation is contagious in all network structures: individuals with a greater fraction of cooperating neighbors are more likely to switch to cooperation themselves.

**Table 1:** The Probability of Switching to Cooperation regressed against % Cooperating Neighbors in Previous Round, without Interactions.

	(1)	(2)	(3)	(4)
	Fixed	Viscous	Fluid	All conditions
% Cooperating Neighbors in Previous Round	0.807**	1.496**	1.479***	1.251***
	(0.332)	(0.661)	(0.502)	(0.259)
Current Round Number	−0.204***	−0.218***	−0.111	−0.154***
	(0.063)	(0.045)	(0.078)	(0.037)
Number of Neighbors in Previous Round	−1.113***	0.272***	0.238***	0.241***
	(0.356)	(0.099)	(0.089)	(0.050)
Number of Neighbors in Current Round	1.038***	−0.171	−0.248***	−0.244***
	(0.364)	(0.158)	(0.063)	(0.056)
Constant	−0.290	−1.545***	−1.312***	−1.107***
	(0.393)	(0.587)	(0.319)	(0.241)
Observations	414	297	299	1,012

Robust standard errors in parentheses.

*** p<0.01,

** p<0.05,

* p<0.1.

This table shows the results from logistic regressions predicting the probability of cooperating in the current round, among individuals who defected in the previous round (i.e. predicting the spread of cooperation). We report the coefficients and robust standard errors clustered on subject and session for each independent variable.

We now ask whether contagion changes over time in each condition by considering the interaction between percentage of cooperative neighbors and round number (Table 2). Doing so, we find no significant interaction in the fixed or viscous conditions, indicating that the spread of cooperative behavior remains constant as rounds progress (fixed coeff = −0.08, *p = *.576; viscous coeff = 0.11, *p* = .685; Table 2 Columns 1 and 2). However, we do find a significant positive interaction in the fluid condition, indicating that, as predicted, the contagion of cooperation grows stronger as rounds progress in the fluid condition (coeff = 0.40, *p = *.025; Table 2 Column 3). To show that this difference between conditions is significant, we perform a regression including data from all conditions, predicting the probability of switching to cooperation. We find a significant three-way interaction between a fluid condition dummy, round number, and percent of cooperative neighbors in the last round (coeff = 0.43, *p* = .040; Table 2 Column 4). Thus, the contagion of cooperation increases significantly more over time in the fluid condition than in the fixed and viscous conditions.

**Table 2:** The Probability of Switching to Cooperation regressed against % Cooperating Neighbors in Previous Round, with Interactions.

	(1)	(2)	(3)	(4)
	Fixed	Viscous	Fluid	All conditions
% Cooperating Neighbors in Previous Round	1.167*	1.018	−0.390	1.082*
	(0.615)	(1.637)	(1.366)	(0.615)
Current Round Number	−0.168**	−0.259***	−0.392***	−0.170***
	(0.074)	(0.089)	(0.096)	(0.056)
Number of Neighbors in Previous Round	−1.120***	0.262**	0.237***	0.208***
	(0.360)	(0.113)	(0.087)	(0.059)
Number of Neighbors in Current Round	1.043***	−0.162	−0.261***	−0.245***
	(0.368)	(0.171)	(0.060)	(0.055)
% Cooperating Neighbors * Round Number	−0.078	0.111	0.402**	−0.018
	(0.139)	(0.273)	(0.179)	(0.131)
Membership in the fluid condition				0.925
				(0.743)
Fluid Condition × % Cooperating Neighbors				−1.494
				(1.387)
Fluid Condition × Round Number				−0.207*
				(0.112)
Fluid Condition × % Cooperating Neighbors × Round Number				0.425**
				(0.207)
Constant	−0.466	−1.338**	0.076	−0.811**
	(0.583)	(0.647)	(0.922)	(0.393)
Observations	414	297	299	1,012

Robust standard errors in parentheses.

*** p<0.01,

** p<0.05,

* p<0.1.

This table shows the results from logistic regressions with interaction terms predicting the probability of cooperating in the current round, among individuals who defected in the previous round (i.e. predicting the spread of cooperation). We report the coefficients and robust standard errors clustered on subject and session for each independent variable, and include interactions between variables.

### Cooperation becomes Contagious in the Fluid Condition as the Network Equilibrates

We now further investigate the emergence of cooperation contagion in the fluid network demonstrated above. We return to analyzing the data from the fluid condition only, and examine the net coefficient ([fraction of C neighbors] + [fraction of C neighbors × round interaction]) to ask in which round cooperation becomes significantly contagious. We find that a marginally significant effect emerges at round 4, and a significant effect emerges at round 5 (net coeff: round three *p* = .334, round four *p* = .072, round five *p* = .002). Consistent with this finding, we see that cooperation is not contagious when aggregating over rounds prior to 5 (Coeff = 1.02, *p* = .297), but is contagious when aggregating over rounds five and greater (Coeff = 2.70, *p*<.0001). This result is consistent with our prediction that contagion should only emerge in the fluid condition once new links are no longer formed frequently (i.e. when the network equilibrates): Figure 1 demonstrates that around round five, the probability of a network update resulting in the formation of a new link levels off and remains relatively low for the duration of the experiment.

We now provide direct evidence for our proposed mechanism, whereby the desire to attract new cooperative neighbors induces players to switch to cooperation regardless of the play of their current neighbors. Our proposed mechanism predicts that contagion increases with round number because round number provides a proxy for perceived ability to attract new cooperative neighbors. If this is the case, then regardless of round number, subjects should be more influenced by their neighbors when they perceive that they have less ability to attract new cooperative neighbors. A clear signal of the current fluidity of the network is the number of new neighbors that an individual gained in the previous round (both through connections others formed with the player, and through connections that the player formed with others). Thus, we predict a negative interaction between number of new neighbors and fraction of cooperating neighbors when predicting the probability of switching to cooperation: subjects’ decisions to switch to cooperation should be less influenced by their neighbors’ previous behavior when more new connections were formed.

To test this prediction, we create a “new connections” variable indicating the number of new neighbors that an individual gained in the previous round, either as a result of choosing to form a connection with another player, or as a result of another player choosing to form a connection with them. We regress the probability of switching to C on the percentage of cooperative neighbors in the previous round, the number of new connections formed in the previous round, the interaction between these variables, and controls for round number and number of neighbors in the current and previous rounds. As predicted, we find a significant negative interaction between the percentage of cooperative neighbors and new connections formed, coeff = −0.59, *p* = .024, indicating that gaining more new connections makes a player’s probability of switching to cooperation depend less on her neighbors’ behavior. This result is consistent with our proposed mechanism, wherein individuals who perceive the opportunity to form new connections in dynamic networks are motivated to switch to cooperation, even when they are paired with relatively uncooperative neighbors. (In contrast, we would not expect a similar relationship to exist for the breaking of connections; and indeed, we find no significant interaction between the percentage of cooperative neighbors and number of connections broken, coeff = 0.09, *p* = .739.).

Thus, while cooperation is contagious across all rounds of the fixed and viscous conditions, it is only contagious in later rounds of the fluid condition. Contagion of cooperation appears to emerge in later rounds of the fluid condition because, as time goes on, the network grows more stable as the probability of forming new connections declines. Therefore, in later rounds fluid networks become functionally similar to fixed and viscous networks, both in terms of the opportunity to form new connections and the contagiousness of cooperation.

### Contagion of Defection

**Figure 3:**
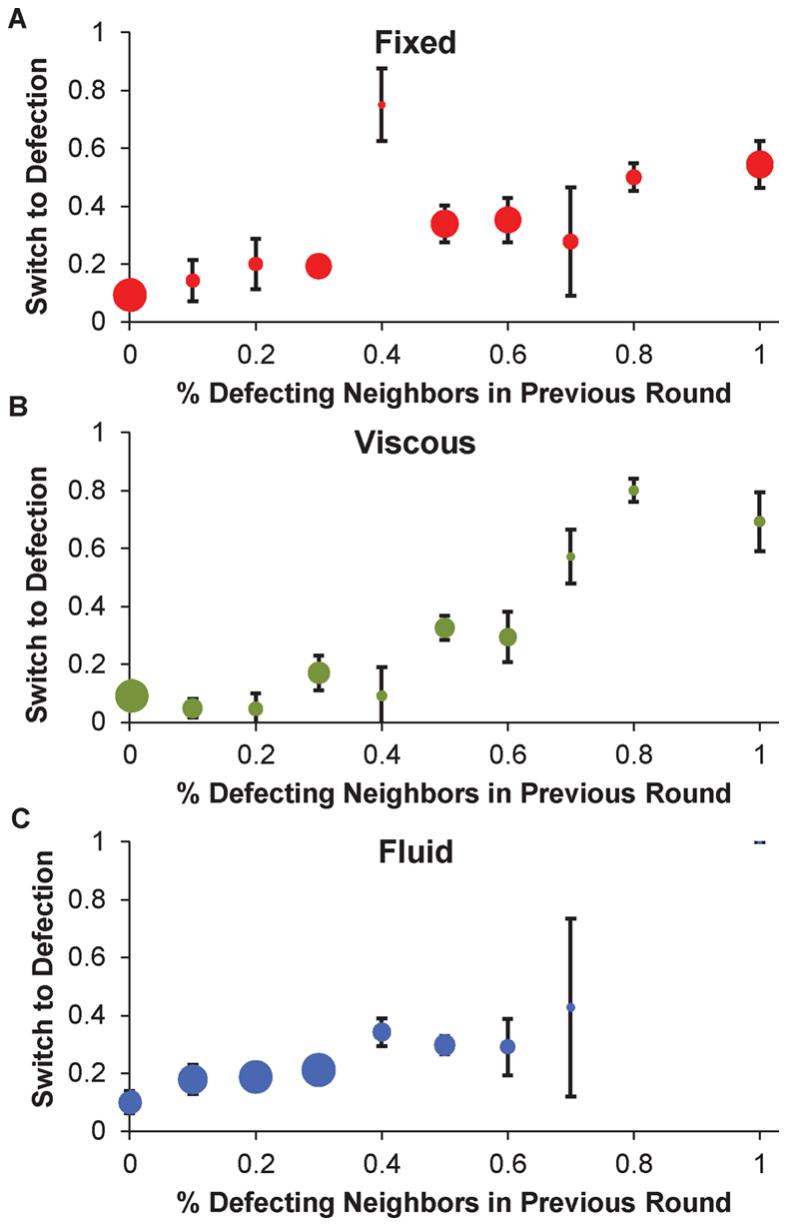
The contagion of defection. This figure shows the probability of switching from cooperation to defection as a function of the percentage of neighbors that defected in the previous round, in the (a) fixed (b) viscous and (c) fluid conditions. Dots depict the percentage of individuals with the specified range of defecting neighbors that switched to defection, and dot size is proportional to the number of observations. Error bars indicate standard error of the mean.

We next consider the spread of selfishness. Figure 3 shows the probability of switching from cooperation to defection as a function of the percentage of defecting neighbors in the previous round for each condition, and Table 3 shows the accompanying regressions. We find a significant negative relationship between the probability of switching to defection and the percentage of cooperating neighbors in each condition individually, as well as collapsed across conditions (fixed coeff = −2.35, *p*<.0001; viscous coeff = −3.63, *p*<.0001*;* fluid coeff = −2.55, *p* = .0001; all conditions coeff = −2.74, *p*<.0001; Table 3 Columns 1–4). Thus, across all rounds of the game, defection is contagious in all network structures: individuals with a greater fraction of defecting neighbors (i.e. a smaller fraction of cooperating neighbors) are more likely to switch to defection themselves.

**Table 3:** The Probability of Switching to Defection regressed against % Cooperating Neighbors in Previous Round, without Interactions.

	(1)	(2)	(3)	(4)
	Fixed	Viscous	Fluid	All conditions
% Cooperating Neighbors in Previous Round	−2.349***	−3.631***	−2.549***	−2.740***
	(0.408)	(0.420)	(0.629)	(0.282)
Current Round Number	0.010	0.026	0.162***	0.060
	(0.034)	(0.083)	(0.041)	(0.039)
Number of Neighbors in Previous Round	−0.365	−0.117	−0.182***	−0.126**
	(0.244)	(0.113)	(0.042)	(0.049)
Number of Neighbors in Current Round	0.462**	0.047	0.115**	0.112***
	(0.231)	(0.104)	(0.048)	(0.035)
Constant	−0.145	1.004**	0.026	0.154
	(0.500)	(0.512)	(0.578)	(0.238)
Observations	377	356	494	1,227

Robust standard errors in parentheses.

*** p<0.01,

** p<0.05,

* p<0.1.

This table shows the results from logistic regressions predicting the probability of defecting in the current round, among individuals who cooperated in the previous round (i.e. predicting the spread of defection). We report the coefficients and robust standard errors clustered on subject and session for each independent variable.

When we consider the interaction between percentage of cooperative and neighbors and round number (Table 4), we find no significant interaction in the fixed or fluid conditions, indicating that the spread of selfish behavior remains constant as rounds progress (fixed coeff = −0.24, *p = *.123; fluid coeff = −0.48, *p* = .184; Table 4 Columns 1 and 3). While we do find a significant negative interaction in the viscous condition (coeff = −1.10, *p* = .0006; Table 4 Column 2), there are no qualitative changes over time: examining the net coefficient shows that selfishness is already significantly contagious in round 2 (the first round in which contagion is possible, *p* = .009), and the interaction merely indicates that the contagion of defection grows stronger as rounds progress.

**Table 4:** The Probability of Switching to Defection regressed against % Cooperating Neighbors in Previous Round, with Interactions.

	(1)	(2)	(3)	(4)
	Fixed	Viscous	Fluid	All conditions
% Cooperating Neighbors in Previous Round	−1.405	0.425	−0.669	−1.225
	(0.919)	(1.254)	(1.127)	(0.765)
Current Round Number	0.090	0.563***	0.494**	0.178**
	(0.057)	(0.104)	(0.243)	(0.079)
Number of Neighbors in Previous Round	−0.371	−0.111	−0.186***	−0.146***
	(0.240)	(0.130)	(0.044)	(0.047)
Number of Neighbors in Current Round	0.464**	0.047	0.113**	0.113***
	(0.225)	(0.125)	(0.046)	(0.040)
% Cooperating Neighbors * Round Number	−0.236	−1.104***	−0.481	−0.417**
	(0.153)	(0.321)	(0.362)	(0.164)
Membership in the fluid condition				−1.028
				(0.840)
Fluid Condition × % Cooperating Neighbors				0.385
				(1.398)
Fluid Condition × Round Number				0.266
				(0.248)
Fluid Condition × % Cooperating Neighbors × Round Number				−0.041
				(0.389)
Constant	−0.486	−1.140	−1.229	−0.236
	(0.576)	(0.889)	(0.793)	(0.459)
Observations	377	356	494	1,227

Robust standard errors in parentheses.

*** p<0.01,

** p<0.05,

* p<0.1.

This table shows the results from logistic regressions with interaction terms predicting the probability of defecting in the current round, among individuals who cooperated in the previous round (i.e. predicting the spread of defection). We report the coefficients and robust standard errors clustered on subject and session for each independent variable, and include interactions between variables.

Thus, contrary to our predictions, we do not find that social contagion of selfish behavior is attenuated in fluid networks. Instead, we find that selfishness spreads from person to person across all rounds of the experiment in all network structures. To further validate this conclusion, we perform a regression including data from all conditions, predicting the probability of switching to defection. We find no three-way interaction between a fluid condition dummy, round number, and percent of cooperative neighbors in the last round (coeff = −0.04, *p* = .915; Table 4 Column 4). Thus, in contrast to the contagion of cooperation, the contagion of defection is not attenuated in early rounds of the fluid condition.

## Discussion

Here we have investigated the contagion of cooperative and selfish behavior in social networks that varied in the extent to which they allowed individuals to update their social network ties. Using a controlled laboratory experiment, we examined changes in behavior over time in fixed, viscous, and fluid networks. While we found that cooperative behavior was consistently contagious in the fixed and viscous network conditions, we found that cooperation only became contagious in fluid networks after the network had equilibrated (i.e. reached a point where new links were formed relatively infrequently). Furthermore, we showed that, in the fluid condition, subjects who gained more new neighbors in a given round were less influenced by the play of their neighbors in the subsequent round. Finally, we found that defection was robustly contagious across all rounds of all network structures. Here, we discuss the implications of three aspects of these results.

Our first key finding is that both cooperation and defection were contagious in fixed networks. This result is partially inconsistent with previous work, which found significant contagion of defection but not cooperation in fixed networks [33]–[35]. This difference may arise from differences in the game payoffs between our experiment and previous studies. Alternatively, this difference may result from the fact that, in previous experiments, subjects were informed of their neighbors’ payoffs after each round as well as their actions. As cooperators earned less than defectors, this payoff information may have discouraged defectors paired with cooperators from switching to cooperation, overwhelming their natural inclination towards mimicry. Further investigation of this issue, for example by manipulating payoff information and holding all other variables constant, is an important direction for future research. Regardless, our data suggest that cooperation can spread from person to person in fixed social networks. This is consistent with the finding that humans are influenced by their social contacts across a wide range of social contexts [15]–[28], and has implications for interventions designed to promote cooperative and other prosocial behavior. In relatively fixed social networks, such as workplaces or apartment buildings, it may be possible for interventions to create cascades of cooperation: increasing cooperation in some target individuals may spread through the network.

Our second key finding is that when social networks afforded the possibility of forming new connections with cooperators, as was the case in early rounds of the fluid condition, the contagion of cooperation was largely attenuated. That is, in early rounds of our fluid condition, subjects were relatively likely to switch from defection to cooperation even when they were paired with uncooperative neighbors. But when social networks did not afford this possibility, as was the case in the fixed and viscous conditions and in later rounds of the fluid condition, subjects reacted to their neighbors, preferentially switching to cooperation when their neighbors were relatively cooperative. We suggest that different network structures create different strategic environments, and thus lead to different strategies for updating our cooperative behaviors in response to those around us: when networks are dynamic, the need to attract cooperative interaction partners suppresses the conditional cooperation strategies employed in static networks, causing players to switch to cooperation regardless of the play of their neighbors.

This finding also has implications for interventions aimed at promoting prosocial behavior via social contagion. In the context of any given application, our results highlight the importance of considering the nature of the social network in question. If it is a fairly fluid network, cooperation may not be contagious. Thus, it may be more effective to attempt to reduce the spread of selfish behavior than to try promoting the spread of cooperative behavior. That is, interventions should aim to keep currently cooperative people from switching to defection, rather than trying to expose defectors to cooperators in an attempt to spread cooperation. However, our results highlight that not all social networks that have the *potential* to be fluid are *actually* fluid at any given time. Dynamic networks can equilibrate, such that even though individuals may have control over their interaction partners, they do not actually wind up forming new connections frequently. Our results suggest that contagion-based interventions should treat such networks liked fixed networks, not fluid networks. We also note that the lack of *contagion* of cooperation in dynamic networks does not imply a lack of *cooperation*: On the contrary, the lack of contagion means that subjects were likely to switch to cooperation even when their neighbors were mostly defecting.

Our third key finding is that unlike the contagion of cooperation, the contagion of defection was not attenuated in dynamic networks. Why might this be? If defectors in the fluid condition are motivated to switch to cooperation to attract new cooperative neighbors regardless of their neighbors’ behavior, then why aren’t cooperators likewise motivated to continue cooperating, even when paired with uncooperative neighbors (as we hypothesized in previous work [42])? It would seem that in early rounds of the fluid condition, instead of mimicking the selfish behavior of one’s neighbors, cooperators would do better to continue cooperating and simply cut ties with defectors when given the chance. Using this strategy, cooperators would be better positioned to attract new cooperative neighbors. However, our data indicate that such cooperators instead switched to defection.

One possible explanation for the asymmetry between the contagion of cooperation and defection comes from the finding that humans have a strong taste for negative reciprocity [6], [8], [50]–[52]. When we perceive that we have been treated unfairly, we are often willing to incur costs to reciprocate the unfair treatment, even when doing so reduces earnings and is thus non-strategic. Negative reciprocity may play a stronger role in the contagion of defection than the contagion of cooperation.

Cooperative players in our game can reciprocate defection either by switching to defection themselves (behavioral reciprocity), or, in the dynamic conditions, by severing their connection with the defector (link reciprocity). Yet even in the fluid condition, only 30% of network connections can be updated each round, and only one member of each pair has control over network updating at any given time. Because the ability to reciprocate by cutting ties is thus limited, behavioral reciprocity may be more psychologically satisfying than waiting for the chance to engage in link reciprocity in the future. Thus, our strong preference for negative reciprocity may contribute to the robust contagion of defection across network structures. In contrast, individuals who have previously defected may be less concerned with avoiding exploitation, and thus less influenced by negative reciprocity motives. It may thus be relatively easier to motivate these individuals to switch to cooperation, even when their neighbors are relatively uncooperative, if they perceive that doing so will attract new cooperative neighbors. This asymmetry could help explain why dynamic networks attenuated the contagion of cooperation, but not the contagion of defection.
